# Development and Characterization of Sustainable Cement-Free Controlled Low Strength Material Using Titanium Gypsum and Construction Waste Soil

**DOI:** 10.3390/ma17235698

**Published:** 2024-11-21

**Authors:** Yunfei Wu, Jian Geng, Haoze Zhu, Chen Jin, Nengneng Kang

**Affiliations:** 1 School of Civil Engineering, Huzhou Vocational and Technical College, Huzhou 313000, China; 2020048@huvtc.edu.cn; 2 School of Civil Engineering, Ningbo Tech University, Ningbo 315100, China; 3 College of Civil Engineering and Architecture, Zhejiang University, Hangzhou 310017, China; 4 Huzhou Zhengtong Traffic Engineering Testing and Inspection Co., Ltd., Huzhou 313000, China

**Keywords:** controlled low strength material, titanium gypsum, construction waste soil, working and hardening propensities, the strength formation mechanism

## Abstract

This study investigates the utilization of titanium gypsum (TG) and construction waste soil (CWS) for the development of sustainable, cement-free Controlled Low Strength Material (CLSM). TG, combined with ground granulated blast furnace slag, fly ash, and quicklime, serves as the binder, while CWS replaces natural sand. Testing thirteen mixtures revealed that a CWS replacement rate of over 40% controls bleeding below 5%, with a water-to-solid ratio between 0.40 and 0.46, ensuring flowability. Higher TG content reduces flowability but is crucial for strength due to its role in forming a crystalline network. Compressive strength decreases with higher TG and water-to-solid ratio, while 3–5% quicklime provides a 56 day strength below 2.1 MPa. Higher CWS reduces expansion, and TG content between 60% and 70% minimizes volume changes. XRD and SEM analyses underscore the importance of controlling TG and quicklime content to optimize CLSM’s mechanical properties, highlighting the potential of TG and CWS in creating low carbon CLSM.

## 1. Introduction

Titanium gypsum (TG) is an industrial byproduct formed during the neutralization of excess waste acid in the production of titanium dioxide via the sulfuric acid method. TG has various utilization strategies, including serving as a cement retarder [1], stabilizer for expansive soil [2,3], CO_2_ sequestration agent [4], adsorbent for wastewater recovery [5], and cementitious material [6]. In China, TG is also employed to build gypsum and improve soil. However, due to its high water content, viscosity, impurity levels, and the presence of ferrous sulfate, the utilization rate of TG remains low. Approximately 22 MMT (million metric tons) of TG are annually generated in China, but only 10% is comprehensively utilized, significantly lower than desulfurization gypsum (80%) and phosphogypsum (40%). Stacking remains the primary disposal method for TG, leading to the long-term occupation of land resources. Additionally, exposure to wind and sunlight causes TG’s fine particles to lose moisture, resulting in dust scattering and environmental pollution.

The Controlled Low Strength Material (CLSM), as defined by the American Concrete Institute (ACI 229R-13, Report on Controlled Low Strength Materials), is a highly fluid and self-compacting material that settles with minimal or no vibration through the force of gravity [7]. It serves as a replacement for traditional backfill materials and typically exhibits an unconfined compressive strength of less than 8.3 MPa after 28 days [8]. For projects involving secondary excavation, the desired strength range is usually between 0.5 and 2.0 MPa [9]. CLSM finds widespread application in backfill engineering, structural filling, road base construction, mine filling, abutments, and other engineering structures. Its use significantly reduces project costs and has gained extensive adoption in many countries [10].

The raw material composition of CLSM resembles that of mortar or general concrete, but it allows for a wider range of binding materials and aggregates with lower quality requirements. It also possesses the ability to incorporate various solid waste materials. Numerous studies have explored the feasibility of using industrial byproducts (such as cement kiln dust, foundry sand, bottom ash, waste glass powder, and wastepaper sludge ash) in CLSM preparation [11,12,13,14]. Additionally, onsite excavated soil is frequently used as an alternative material for CLSM production. Qian and Hu et al. evaluated the performance of CLSM with excess excavation partially replacing sand, revealing that increased soil content resulted in reduced strength, flowability, water stability, and frost resistance, while shrinkage was superior to most cement soils [15]. Sheen and Zhang et al. found that low liquid limit soil-based CLSM exhibited good excavation performance, and the flowability could be enhanced by partially replacing cement with blast furnace slag [16]. Puppala and Chittoori et al. successfully prepared CLSM using high plastic soil for pipe trench backfilling projects, achieving positive economic and environmental outcomes [17]. Based on the aforementioned findings, it can be concluded that incorporating an appropriate proportion of soil into CLSM is entirely feasible.

Traditionally, Portland cement has been the primary binding material used in the preparation of CLSM. However, the production of cement generates significant greenhouse gases such as carbon dioxide, sulfur oxide, and nitrogen oxide. To address this concern, alkali-activated cementitious materials have emerged as a promising alternative to replace cement in CLSM production [18,19,20]. These materials, also known as alkali-activated silica-aluminum salts, have the advantage of producing significantly lower carbon dioxide emissions compared to ordinary Portland cement during their production process. They are typically formed through the polymerization reaction of volcanic ash materials like fly ash and metakaolin with alkaline activators.

In summary, while there exists a variety of raw materials for preparing CLSM, there is a lack of reports regarding the use of TG in CLSM preparation. Additionally, current Controlled Low Strength Material (CLSM) binder materials predominantly consist of Portland cement. However, considering the low requirement for raw material quality in CLSM and the need for the development of low carbon civil engineering materials, non-cementitious-based CLSM should be the main direction for its future development. On one hand, this would help reduce its cost, and on the other hand, it would facilitate the resource utilization of solid waste.

This study aims to evaluate the feasibility of utilizing a composite of titanium gypsum (TG) and construction waste soil (CWS) for the production of cement-free Controlled Low Strength Material (CLSM), with a focus on innovatively replacing traditional materials to develop more sustainable construction practices. TG, used in its dihydrate form without dehydration treatment, along with ground granulated blast-furnace slag (GGBS) and fly ash, serves as the primary cementitious material, while quick lime is employed as an activator. CWS is explored as a substitute for natural river sand to assess its impact on the engineering performance of CLSM. This study thoroughly investigates the effects of varying CWS replacement rates, water-to-solid ratios, TG content, and activator content on CLSM’s properties, including setting and hardening mechanisms analyzed through X-ray Diffraction (XRD) and Scanning Electron Microscopy (SEM). This research contributes novel insights into the potential of TG and CWS in developing low carbon, resource-efficient construction materials, addressing the pressing need for sustainable alternatives in civil engineering.

## 2. Materials and Methods

### 2.1. Raw Materials

**Figure 1:**
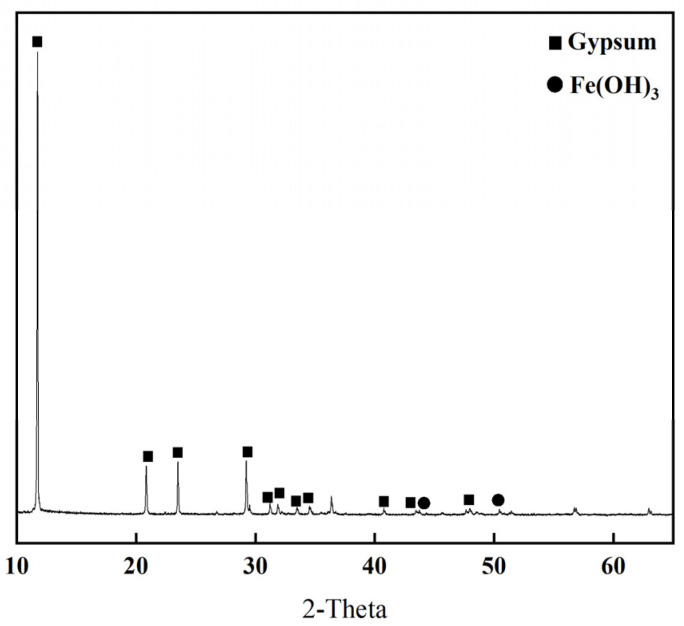
XRD pattern of TG.

Titanium gypsum was obtained from the industrial waste of a local titanium dioxide factory in Ningbo, China. Initially, this TG was a powdery solid, with moisture content fluctuating between 30% and 50%, and exhibited a brownish-yellow or reddish color. For experimental preparation, the TG was subjected to drying, crushing, and screening processes at a low temperature of 50 °C, resulting in a finely powdered form with a specific surface area of 530 m^2^/kg. As depicted in Figure 1, the XRD analysis identified the predominant crystal phase in the TG as dihydrate gypsum, accompanied by Fe(OH)_3_ impurities.

The ground granulated blast furnace slag (GGBS) used in this study was sourced from a steel plant. It is a gray-white powdery solid obtained through grinding and possesses a specific surface area of 425 m^2^/kg. The fly ash was collected from the Ningbo Beilun power plant and is classified as grade II, with a specific surface area of 480 m^2^/kg. The quicklime was obtained from Sinopharm Reagent Co., Ltd., and has an effective CaO content of 98%. Table 1 provides the chemical compositions of all the main raw materials used in this study.

**Figure 2:**
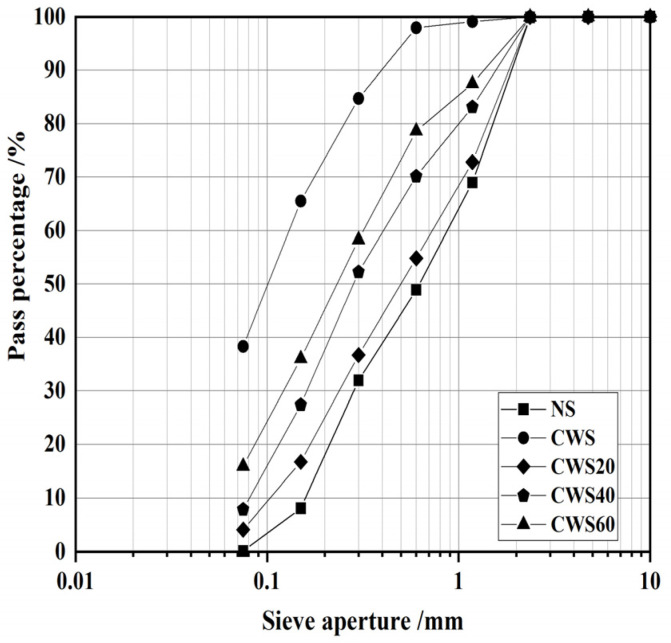
Cumulative particle size distribution of NS and CWS.

The fine aggregate used in this experiment consists of two components: natural river sand (NS) and CWS. The fineness modulus of the natural river sand that has not been cleaned is 2.42. The CWS was obtained from a construction site in Ningbo, China. The debris, such as tree branches, plastics, and large sized crushed stones, was removed. After being crushed by a jaw crusher and dried at 105 °C until a constant weight was reached, the CWS was prepared for the next step. Subsequently, the CWS was sieved through a 4.75 mm square hole sieve. Table 2 presents the basic physical properties of the CWS. Figure 2 illustrates the cumulative particle size distribution of both the NS and CWS. It is evident that the CWS has a higher proportion of fine particles compared to natural sand. By replacing a portion of the NS with CWS, the particle size distribution of the fine aggregate was altered. Increasing the replacement rate of CWS from 0% to 60% leads to a gradual decrease in the overall particle size of the fine aggregate.

### 2.2. Test Mix Ratio

In this study, a mass ratio of 3:1 was set for fly ash to GGBS, while the binder-aggregate ratio (i.e., the mass ratio of binder to fine aggregate) was set to 1:1. Utilizing the replacement rate of CWS, water-to-solid ratio, TG content, and quicklime content as mix proportion parameters, a total of 13 groups CLSM were designed using the control variable method. Within these CLSM samples, the replacement rates of CWS were varied at 0%, 20%, 40%, and 60%, while the water-to-solid ratios were adjusted to 0.40, 0.42, 0.44, and 0.46. Additionally, the content of TG ranged from 40% to 70%, and the content of quicklime varied between 3% and 9%. The details of the mixed proportion of each sample are shown in Table 3.

### 2.3. Test Methods

**Figure 3:**
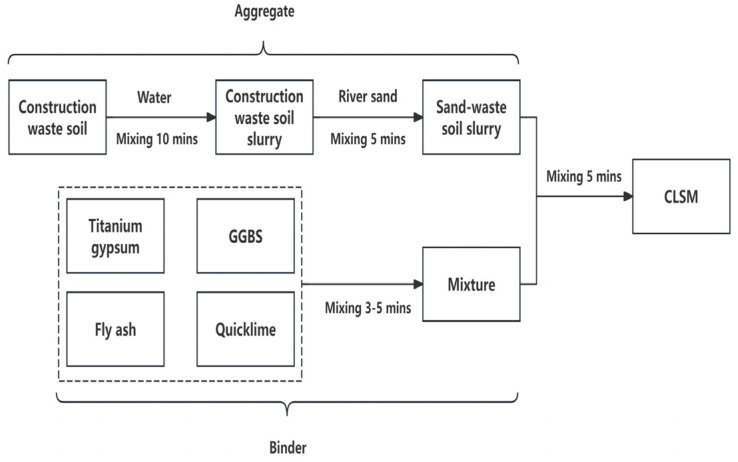
CLSM preparation process.

The preparation process, which is based on extensive indoor pretests, is outlined in Figure 3. Flowability and bleeding rate are two key performance indicators for CLSM. The flowability test was conducted in accordance with ASTM D6103-17 [21] using an open cylindrical mold with dimensions of 75 × 150 mm. The bleeding rate was measured for freshly mixed slurry using a 1 L container, following the guidelines of ASTM C940-16 [22]. For evaluating the unconfined compressive strength, cubic blocks measuring 70.7 × 70.7 × 70.7 mm, as specified in JTG 3420-2020 [23], were cast and molded. Due to the lower early strength of the CLSM, the test specimens were demolded after being cured for 2 days. These specimens were then placed in a standard curing room at a temperature of (20 ± 2) °C for 3, 7, 14, 28, and 56 days. The loading rate during the tests was controlled at 1 mm/min. To analyze the hydration products and micro structural characteristics of hardened CLSM, the hydration process was terminated using anhydrous ethanol. The specimens were then dried at 45 °C until a constant weight was achieved for subsequent XRD and SEM analysis.

In the volume stability test that was conducted in accordance with the Chinese standard JGJ/T 70-2009 [24], specimens measuring 40 × 40 × 160 mm were utilized. Copper nails were inserted at both ends of the specimens to facilitate the measurement of length changes at time intervals of 4, 5, 7, 9, 11, 14, 17, 21, 28, and 56 days. The measurements were conducted on three sets of parallel specimens to ensure the accuracy and reliability of the test results.

In order to evaluate the stiffness properties of CLSM material, a cylinder specimen of ø 50 × 100 mm was used to measure the stress-strain change process of CLSM under load after standard curing for 28d according to the Chinese standard JGT E40-2007 [25]. Then, the slope of the line at the point corresponding to the origin of the stress-strain curve and the peak stress 0.5 times were used as the elastic modulus [26,27].

## 3. Test Results and Discussion

### 3.1. Flowability and Bleeding

**Figure 4:**
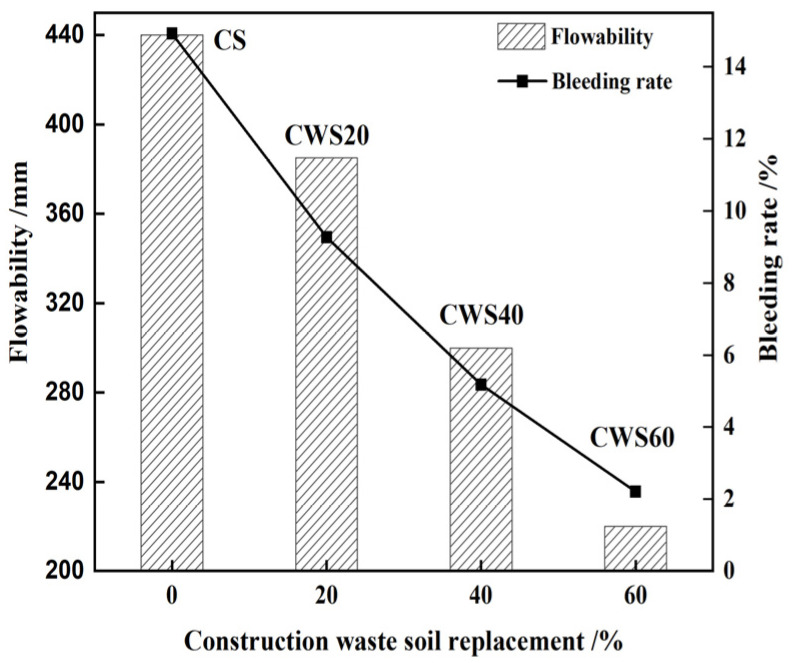
Influence of S/A on the flowability and bleeding rate of CLSM.

**Figure 5:**
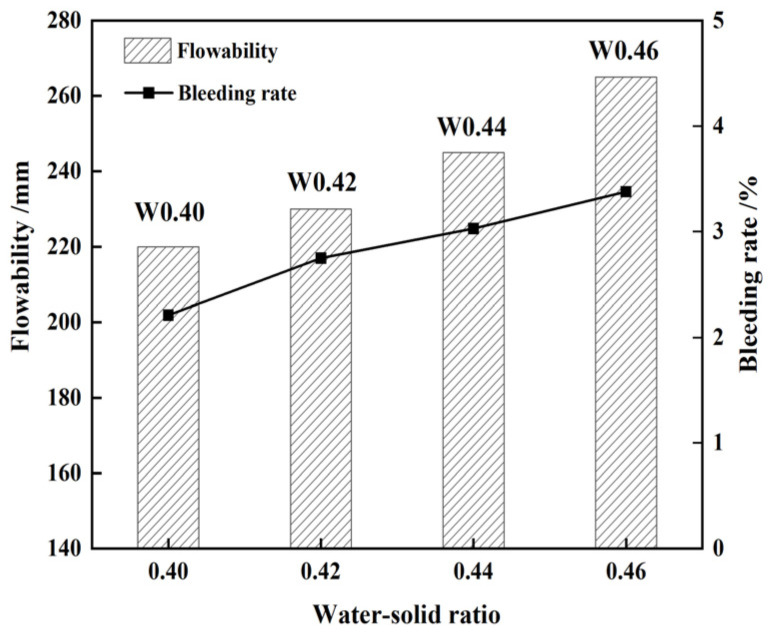
Influence of W/(B + A) on the flowability and bleeding rate of CLSM.

**Figure 6:**
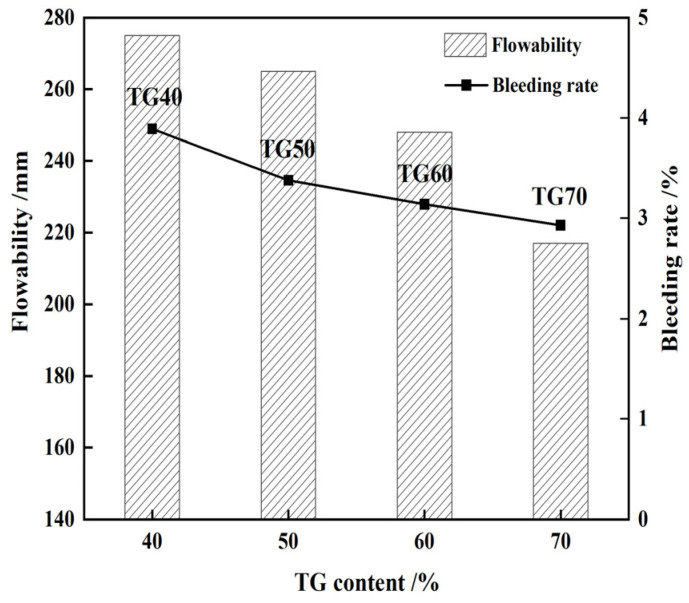
Influence of TG content on the flowability and bleeding rat of CLSM.

**Figure 7:**
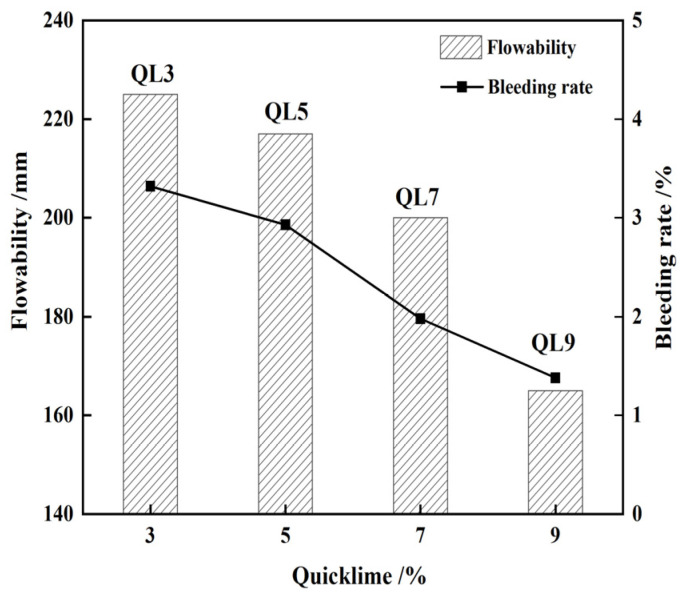
Influence of quick lime content on the flowability and bleeding rat of CLSM.

According to the ACI-229R guidelines, the optimal flowability range for CLSM should be maintained between 200 and 300 mm [7]. Additionally, the flowability should not be less than 180 mm to ensure adequate slurry self-filling and self-compaction [28,29]. The figures depicted in Figure 4, Figure 5, Figure 6 and Figure 7 demonstrate that the minimum flow requirements were met for all test groups except for sample QL9, which contained 9% quicklime. It is worth noting that achieving better flowability in CLSM often requires a significant amount of mixing water. However, excessive free water can lead to bleeding, as it may be absorbed by surrounding media or precipitated from the surface. This can result in segregation and an uneven distribution of various components, making it challenging to maintain the strength of the slurry. To address this issue, it is generally recommended, as suggested by Lachemi and Dickson et al. [30,31], to control the maximum bleeding rate of CLSM below 5%. and Kim attributed it to the stable CLSM formulations [32]. This helps to minimize the negative effects of excessive water content, ensuring better performance and maintaining the desired characteristics of the CLSM.

Figure 4 illustrates a significant decrease in the flowability of CLSM as the value of S/A (i.e., the replacement rate of CWS to NS) increases. On the other hand, the bleeding rate decreases with the increase in the replacement rate of CWS. When the replacement rate reaches 40% or higher, the bleeding rate can be controlled within 5%. Xiao’s research on preparing CLSM with waste glass and red mud indicated that the bleeding rates of most samples were less than 5%, and red mud had a good controlling effect on it [12]. For this research, as the CWS content increases, the NS content decreases, leading to an increase in the specific surface area of the mixture. Consequently, more water is required to coat the particle surface of the CWS. Moreover, the residue from CWS exhibits certain cohesive features. Therefore, under the same W/(B + A) (i.e., water-to-solid ratio), the CLSM bleeding rate reduces with the increase in CWS content.

It is observed that when the W/(B + A) increases from 0.4 to 0.46, as shown in Figure 5, both the flowability and bleeding rate of the CLSM increase, which is consistent with the research findings of Zhao, Khadka, and Lee et al. [20,33]. This is due to the fact that the increased water-to-solid ratio exceeds the water consumption required for the hydration reaction of raw materials. The excess mixing water fills the pores between particles and forms a water film on the particle surfaces.

Figure 6 demonstrates the impact of TG content on the working performance of CLSM. It is found that as the TG content increases under the same water-to-solid ratio, the flowability and bleeding rate of the CLSM gradually decreases. This is attributed to the larger specific surface area of TG compared to mineral powder and fly ash, which results in higher adsorption of free water. As a result, the slurry with a higher TG content exhibits higher consistency and lower flowability.

From Figure 7, it is observed that the flowability of the corresponding CLSM gradually decreases as the amount of quicklime increases from 3% to 9%. When the quicklime content exceeds 5%, the flowability of the CLSM experiences a significant decrease. Particularly, when the quicklime content reaches 9%, the flowability drops to 165 mm, representing a decrease of approximately 26.7%.

**Figure 8:**
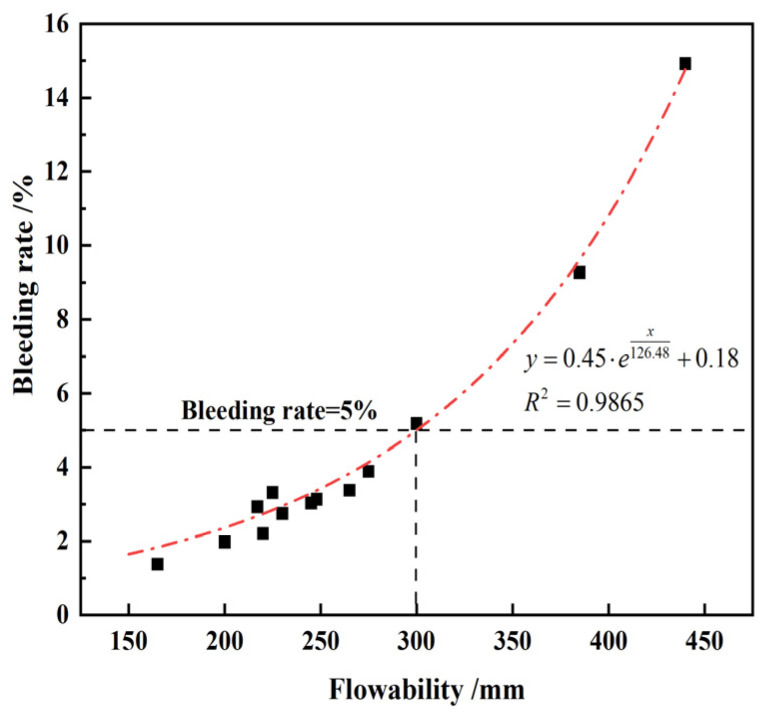
Relationship between bleeding rate and flowability based on 13 distinct mix ratio tests.

The flowability and bleeding rate of fresh CLSM were examined across 13 distinct mix ratio tests, with their interrelation depicted in Figure 8. The figure reveals that the change pattern of CLSM flowability is generally consistent with the change pattern of the bleeding rate under various influencing factors. An increase in the CLSM flowability corresponds to an increase in the bleeding rate, indicating reduced resistance to bleeding of the CLSM. As a result, in practical engineering applications of CLSM, both flowability and bleeding rate should be considered. Based on the findings presented in Figure 8, it is recommended to control the upper limit of flowability for CLSM composed of TG below 300 mm to achieve a bleeding rate below 5%. This ensures that the slurry meets the requirements and maintains the desired performance during application in engineering projects. It should be noted that the bleeding property has a strong correlation with the raw material composition of CLSM. Aggarwal prepared CLSM using foundry sand and cement kiln dust. Their results demonstrated that when the bleeding rate was less than 5%, the fluidity was no greater than 240 mm [14].

### 3.2. Unconfined Compressive Strength

Controlled Low Strength Material differs from traditional compacted soil in the principle of strength development [34,35]. Traditional compacted soil typically gains strength by compaction, which reduces voids and expels pore water, thereby increasing the unit weight of the soil and forming a relatively dense and cohesive mass. In contrast, the construction of CLSM only requires direct pouring without the need for complex processes such as rolling and tamping. The strength of CLSM primarily arises from various physical-chemical reactions occurring between the raw materials of the mixture, which harden to form an integrated structure and generate strength. The mechanism of strength formation is similar to that of soil reinforcement with cement [6,36].

**Figure 9:**
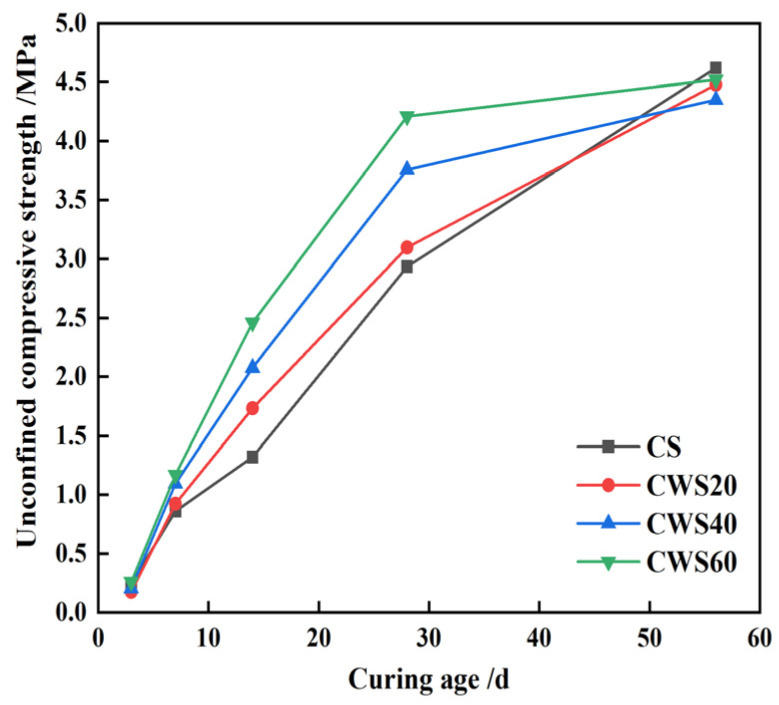
CLSM strength variation characteristics under different replacement rates of CWS.

Figure 9 demonstrates that the compressive strength of CLSM increases with higher S/A values within 28 days. Beyond this period, strength development decelerates as the replacement rate of CWS rises. At 28 days, the CS and CWS60 show compressive strengths of 2.9 and 4.2 MPa, respectively, with a 1.3 MPa difference. By 56 days, this difference reduces to 0.1 MPa. The growth rates of compressive strength for CS and CWS60 from 28 to 56 days are 57.7% and 7.4%, respectively. Thus, replacing natural sand with CWS boosts early CLSM strength but slows later strength development. It should be mentioned that Zhu found that when using sandy soil to prepare CLSM, due to continuous hydration and pozzolanic reactions, the strength of CLSM still increased significantly from 28 to 56 days because the cementitious materials were cement and fly ash [37]. According to ACI 229R, this slower development is beneficial for future secondary excavation of CLSM.

**Figure 10:**
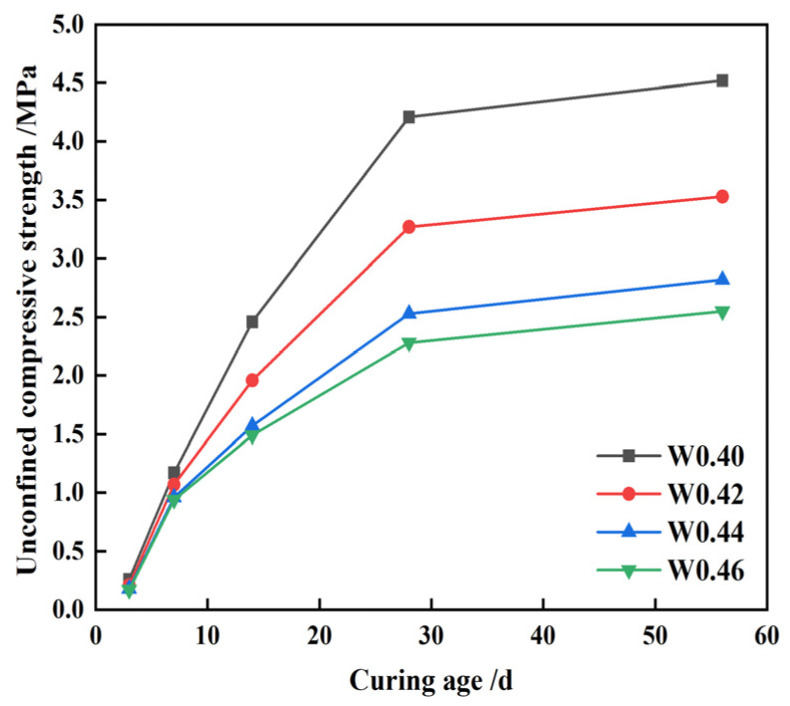
CLSM strength variation characteristics under different water-to-solid ratios.

When the replacement rate of CWS is fixed at 60%, with constant quicklime content at 5% and TG at 50%, increasing the water-to-solid ratio (W/(B + A)) from 0.40 to 0.46 results in 56 day compressive strengths of 4.5, 3.3, 2.5, and 2.3 MPa, respectively, as shown in Figure 10. This demonstrates that the compressive strength of CLSM decreases with a higher water-to-solid ratio. The underlying reason is that while water facilitates reactions such as hydration, ion exchange, and aggregation, excess water beyond the required amount does not participate in these reactions promptly. Instead, it remains in the mixture, forming numerous internal pores, which weakens the strength of the CLSM specimen.

**Figure 11:**
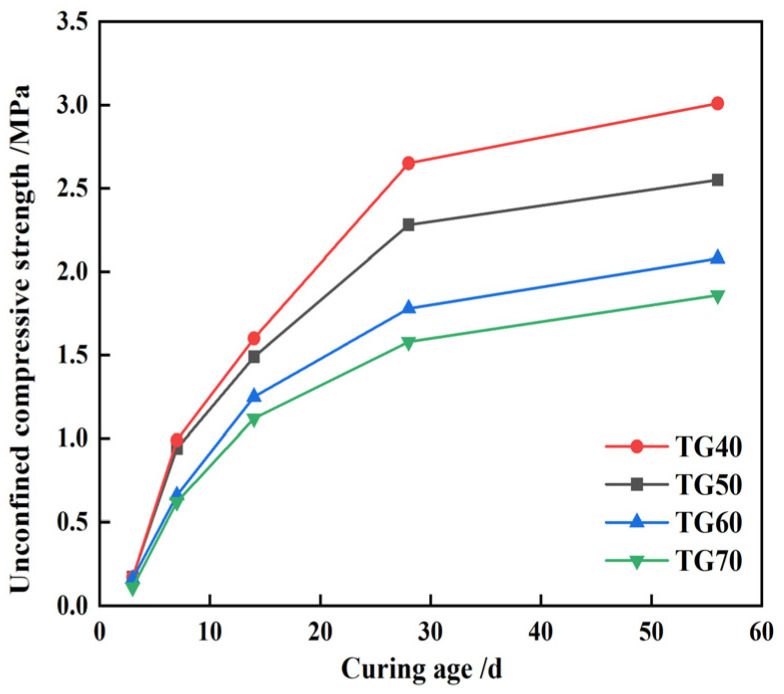
CLSM strength variation characteristics of different TG content.

Figure 11 shows that the compressive strength of CLSM decreases as the TG content increases. At 40% TG, the CLSM strength at 28 and 56 days is 2.7 and 3.0 MPa, respectively. When the content rises to 70%, the strength drops to 1.6 MPa and 1.9 MPa, reflecting decreases of 40.4% and 38.2%. Thus, higher TG content effectively reduces the compressive strength of CLSM. Similar results were also reported by Wang, showing that the compressive and flexural strengths of the self-leveling mortar reached the highest when the content of titanium gypsum was 45% [38]. However, Lin once studied the influence of the Titanium gypsum-based stabilizer (TS) on the unconfined compressive strength of silt. The results showed that as the content of TS increased, the strength of silt also increased. This is somewhat different from the results in this paper. The reason is that TS is composed of cement, lime, and titanium gypsum, and the contribution of cement to strength is higher than that of titanium gypsum [3].

**Figure 12:**
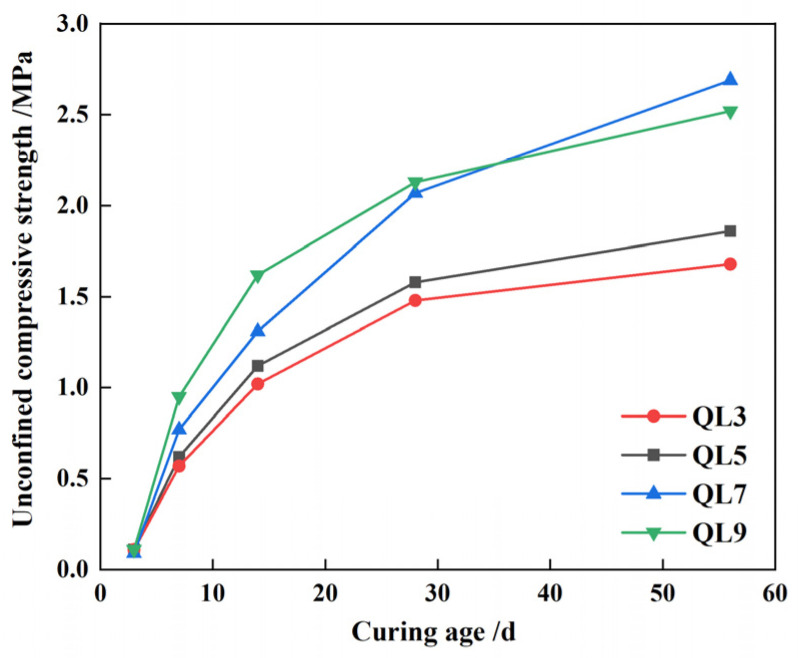
CLSM strength variation characteristics under different content of quicklime.

The compressive strength of CLSM at 56 days increases by 0.2, 0.3, 0.6, and 0.4 MPa as quicklime content rises from 3% to 9%, compared to the strength at 28 days. This indicates that higher quicklime content promotes later-stage compressive strength growth by maintaining system alkalinity and ensuring the presence of Ca(OH)_2_ in the pore solution. This environment supports the continuous dissolution of the glass phase in fly ash and GGBS. At 56 days, samples with 3% and 5% quicklime content show strengths of 1.7 and 1.9 MPa, respectively, with slower growth rates. A compressive strength below 2.0 MPa is advantageous for later secondary excavation, suggesting that quicklime content should not exceed 5%. These findings are illustrated in Figure 12.

### 3.3. Volume Stability

Volume stability is an important indicator for assessing the engineering properties of CLSM. Excessive expansion or shrinkage of the material can cause cracking, thereby shortening its lifespan.

**Figure 13:**
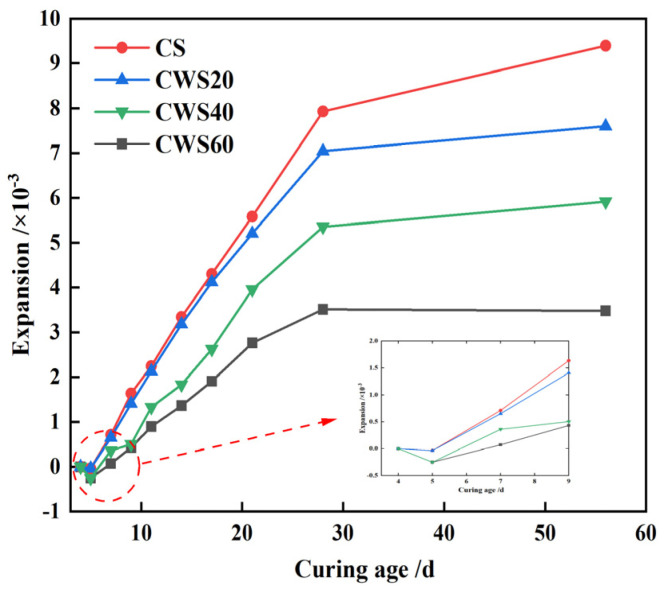
Volume change characteristics of CLSM under replacement rates of CWS.

The expansion rate of CLSM decreases as the replacement rate of CWS for natural sand (S/A) increases, as depicted in Figure 13. For instance, CS has an expansion rate of 0.07% at 7 days, increasing to 0.94% at 56 days. In contrast, the expansion rate of CWS60 is negligible at 7 days but reaches 0.35% at 56 days. Moreover, except for CS, the expansion of other samples stabilizes by 28 days. These findings suggest that adding CWS effectively limits the expansion characteristics of CLSM. This effect may be due to the water loss shrinkage of CWS, which reduces the volume expansion caused by ettringite formation during curing.

**Figure 14:**
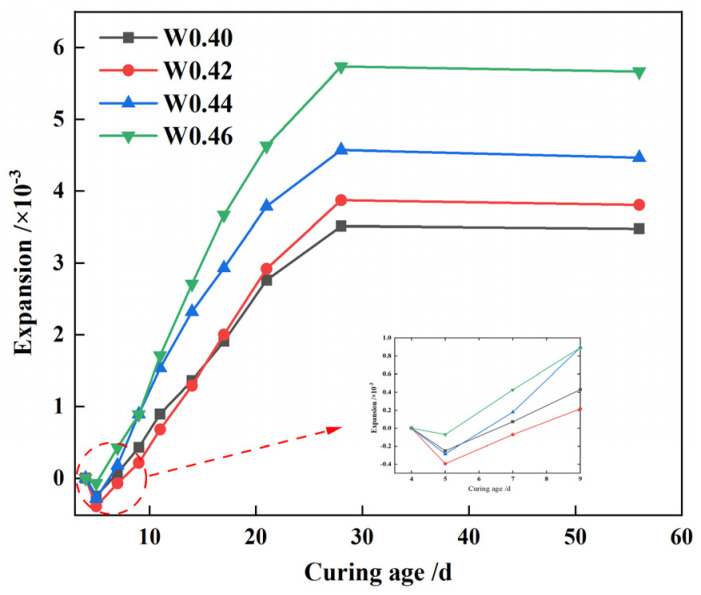
Volume variation characteristics of CLSM under different water-to-solid ratios.

Figure 14 illustrates that the expansion rate of CLSM rises with an increase in the water-to-solid ratio. At a ratio of 0.40, the CLSM shows a 56 day expansion rate of 0.4%. Yet, with an increased ratio of 0.46, the expansion rate escalates to 0.6%, marking an almost 60% increase. This phenomenon occurs because a higher water-to-solid ratio creates more pores in the CLSM, enlarging the spacing between particles and weakening the bonding force within the hardened body. At elevated ratios, the expansion force from hydration, leading to the formation of ettringite and calcium hydroxide crystal, surpasses the bonding force, resulting in increased CLSM volume expansion.

**Figure 15:**
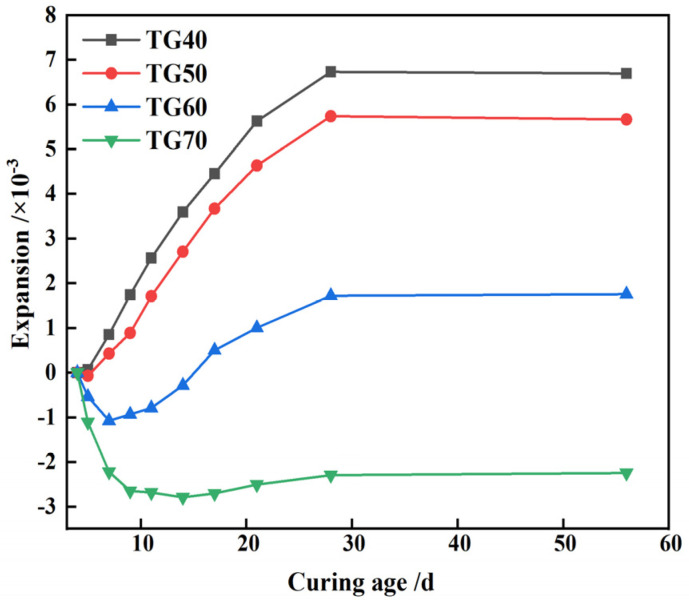
Volume change characteristics of CLSM under different content of TG.

Figure 15 highlights a notable decrease in CLSM expansion rates with increasing TG content. Within the 40% to 50% range, the CLSM volume tends to expand. However, at 60% content, initial shrinkage followed by expansion occurs. At 70%, the CLSM volume trends towards shrinkage, peaking at 0.27% after 14 days. This decrease likely stems from reduced ettringite formation due to declining GGBS and fly ash content. Consequently, early expansion in CLSM becomes less pronounced compared to water-loss shrinkage in CWS, resulting in overall shrinkage. Moreover, higher TG and CWS content delays GGBS and fly ash hydration, posing crack risks. To manage expansion or shrinkage risks, controlling the TG content within 60% to 70% is advisable.

**Figure 16:**
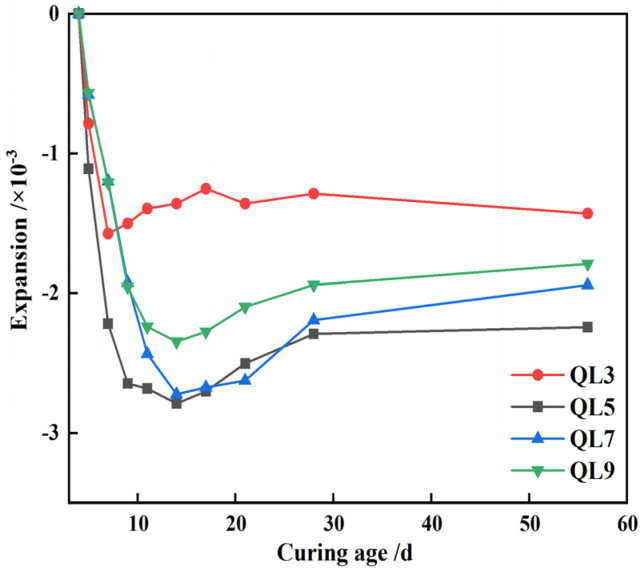
Volume variation characteristics of CLSM under different content of quicklime.

Based on the information presented in Figure 16, which focuses on a high TG content of 70%, the shrinkage rate of CLSM at 56 days exhibits a pattern where it initially increases and then decreases with an increasing content of quicklime. However, even when the quicklime content surpasses 5%, the volume of CLSM continues to exhibit a shrinking trend during the later period. It should be pointed out here that the research results of Mneina [38] and Ghanad [39] showed that the contraction of CLSM is generally in the range of 0.02% to 0.08%, which is significantly lower than the experimental results in this paper. The reason for this is that the hardening properties of CLSM are highly related to the composition of raw materials. The raw materials used by Mneina and Ghanad were all cement and fly ash, and the aggregate was river sand with a higher stiffness than CWS.

### 3.4. Elastic Modulus

The stress-strain curve can reflect the state of force applied to a material and is the most fundamental index for describing the mechanical properties of a material. Therefore, investigating the stress-strain variation of CLSM under load, as well as the process leading to failure, is highly significant for a thorough understanding of the mechanical properties of CLSM based on TG and CWS. In this section, under the conditions of a fixed lime content of 5% and a water-to-solid ratio of 0.46, stress-strain curves were plotted for samples with different TG contents. By combining the plotted stress-strain curves, an analysis of the stiffness characteristics of CLSM based on TG and CWS was conducted.

**Figure 17:**
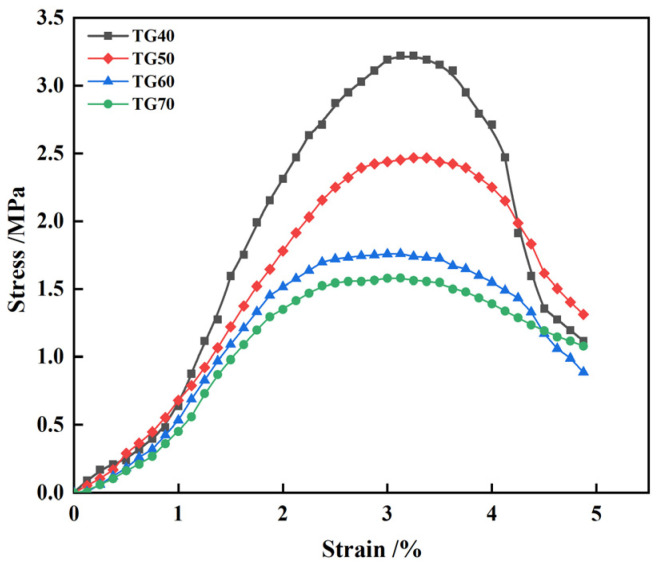
Stress-strain curves of CLSM samples with different TG content.

Figure 17 shows the stress-strain curve of CLSM samples with different TG content after 28 days of curing; it shows that the content of TG has a great influence on the stress-strain curve of CLSM. However, the curves are composed of rising and falling sections. The slope of the rising section of the stress-strain curve gradually increases, and the descending section becomes steeper with the decrease of the content of TG. When the TG content is greater than 60%, the strain corresponding to the stress peak is about 2% to 4%, which is similar to the stress growth curve of ordinary clay. When the peak stress approaches, the stress growth slows down and remains stable for a long time, showing certain ductility characteristics. When the TG content is between 40% and 50%, the initial stress increases rapidly, and the stress decreases rapidly after reaching the maximum failure stress, and the opening of the stress-strain curve becomes narrower, indicating that the brittleness of the material increases.

The calculation results of the CLSM elastic modulus under different TG content are listed in Table 4. It shows that the CLSM elastic modulus decreases with the increase of TG content. This result is similar to that of Halmen et al., who prepared CLSM with synthetic gypsum and pointed out that excessive gypsum may lead to expansion cracks in CLSM and reduce its elastic modulus [39]. As Table 4 shows, the value of elastic modulus ranges from 60.6 to 106.5 MPa. At the same time, the elastic modulus of general subgrade soil filler is between 30 and 60 MPa [40]. In practical engineering applications, a higher elastic modulus can reduce the deformation after construction and the pressure exerted by moving traffic load on pipelines, which is conducive to improving the stress status of pipelines in trench backfilling engineering [41]. In addition, the table shows that the ratio of elastic modulus to 28 days compressive strength is stable at about 33. That is, E50 is approximately 33 times of 28 days compressive strength. The elastic modulus increases with the compressive strength of CLSM, and there is a good correlation between them. Zhao’s results show that there is a linear relationship between the CLSM elastic modulus and the compressive strength [27]. Therefore, the compressive strength can be used to predict the elastic modulus of CLSM.

Table 5 presents the experimental results of peak strain and ultimate strain. It can be observed from the table that for samples with different TG contents, the peak strain at the age of 28 days ranges from 29.38 × 10^−3^ to 33.37 × 10^−3^, and the ultimate strain ranges from 40.01 × 10^−3^ to 41.65 × 10^−3^. In engineering, the ratio of ultimate strain to peak strain is commonly used to characterize the ductility of a material. The calculated results from the table show that there is a certain regularity in the ratio of ultimate strain to peak strain, which gradually increases with the increase of TG content and is all less than 1.5, indicating plastic failure.

### 3.5. The Strength Formation Mechanism of CLSM

#### 3.5.1. The Hydration Process

**Figure 18:**
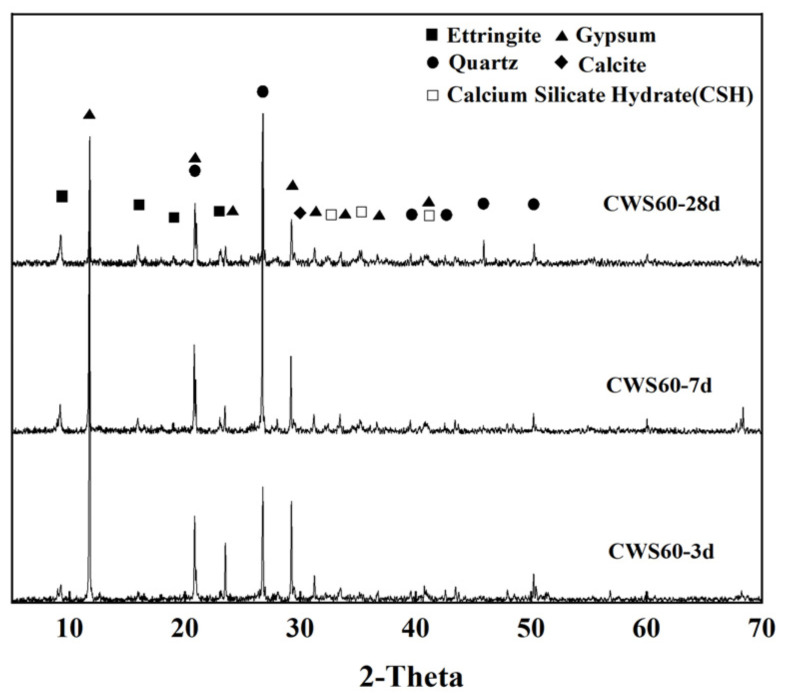
XRD patterns of CWS60 samples at different ages.

Figure 18 shows the XRD patterns of CWS60, which has superior mechanical properties compared to the other samples, at curing ages of 3, 7, and 28 days. As seen from the figure, the hydration products primarily consist of ettringite, calcium silicate hydrate (C-S-H gel), residual dihydrate gypsum, and quartz introduced with the raw materials. The diffraction peak of ettringite appears as early as 3 days, indicating the formation of ettringite crystals at this stage. The intensity of the ettringite diffraction peak continues to increase with curing time, showing a growth trend even at 28 days of hydration. This suggests that ettringite in CLSM continues to form up to 28 days of hydration.

Additionally, the XRD patterns indicate the change in the diffraction peak of dihydrate gypsum with curing time. The intensity of the gypsum diffraction peak decreases continuously as the curing period progresses, indicating the ongoing consumption of gypsum during the hydration process. However, a significant amount of residual gypsum is still present in the hardened CLSM at 28 days. Under the action of OH^−^, the silicon aluminum vitreous phase in GGBS and fly ash continuously dissolves, generating ions like [SiO_4_]^4−^ and [AlO_2_]^−^. These ions react with Ca^2+^ and SO_4_^2−^ in the liquid phase to form calcium silicate hydrate and ettringite, which are of crucial importance for improving the mechanical properties of CLSM [12,38,42].

**Figure 19:**
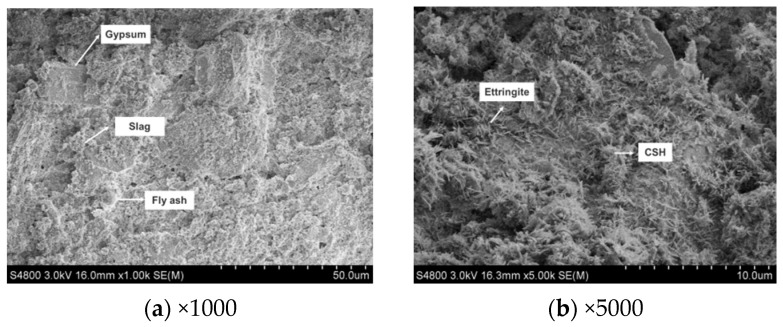
The SEM images of T4 at 3 days.

**Figure 20:**
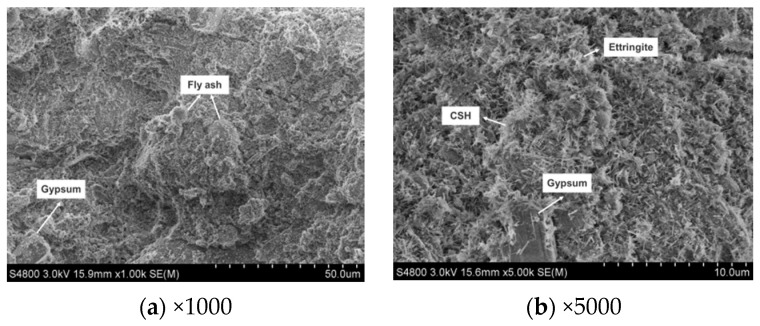
The SEM images of CWS60 at 7 days.

**Figure 21:**
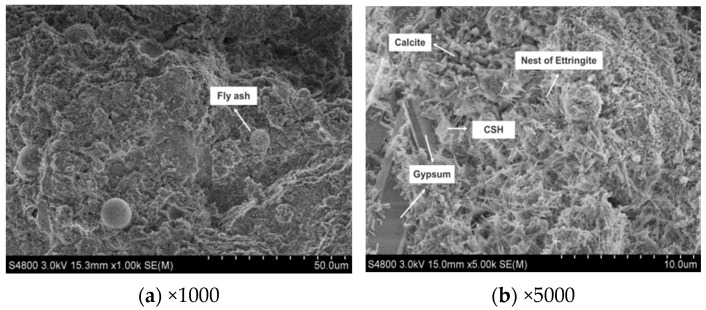
The SEM images of CWS60 at 28 days.

Figure 19, Figure 20 and Figure 21 show the SEM images of the hydration products of CWS60 samples at 3, 7, and 28 days. From Figure 19, at 3 days, the structure appears loose, dominated by calcium silicate hydrate gel and needle-like ettringite crystals. With the increase in the curing age, the glassy phases in mineral powder and fly ash are continuously dissolved and hydrated. Meanwhile, the active colloidal Al_2_O_3_ and SiO_2_ in the clay minerals of CWS will also have chemical reactions with quicklime, generating more calcium silicate hydrate and calcium aluminate hydrate gels.

Compared to the microstructure at 3 days, the sample at 7 days exhibits a relatively denser internal structure with an increased quantity of ettringite crystals, as depicted in Figure 20. The generated ettringite and calcium silicate hydrate gel intertwine and gradually fill the pores, further enhancing the compactness of the hardened paste [43]. Additionally, residual reacted TG appears as thick plate-like crystals interspersed within the structure, contributing to a supportive framework.

From Figure 21, it is evident that at 28 days of hydration, the quantity of ettringite continues to increase, forming a dispersed cluster-like crystalline network with larger crystal sizes. The dense intergrowth and cross-linking between ettringite crystals and calcium silicate hydrate gel not only densify the hardened structure but also provide internal framework support. Additionally, hydration products encapsulate unreacted TG, fly ash, and GGBS particles. Furthermore, the presence of a small amount of granular calcite is observed in the image, possibly resulting from the carbonation of calcium hydroxide.

Based on the XRD and SEM analyses of CWS60 samples at 3, 7, and 28 days, the setting and hardening processes of CLSM based TG and CWS can be summarized as follows: Upon mixing CLSM components with water, quicklime reacts with water to form Ca(OH)_2_. GGBS and fly ash begin to dissolve and hydrate under the alkaline activation of Ca(OH)_2_. As glass phases in GGBS and fly ash dissolve and some TG dissolves in water, the crystallization of ettringite begins in the liquid phase, accompanied by the formation of amorphous calcium silicate hydrate gel. With increasing curing time, continuous hydration of GGBS and fly ash, along with chemical reactions between Ca(OH)_2_ and reactive clay mineral components, lead to the formation of more reaction products. Numerous rod-shaped ettringite crystals intersperse and bond together to form a crystalline network, providing the foundation for the strength development of hardened CLSM.

#### 3.5.2. The Effect of TG Content

**Figure 22:**
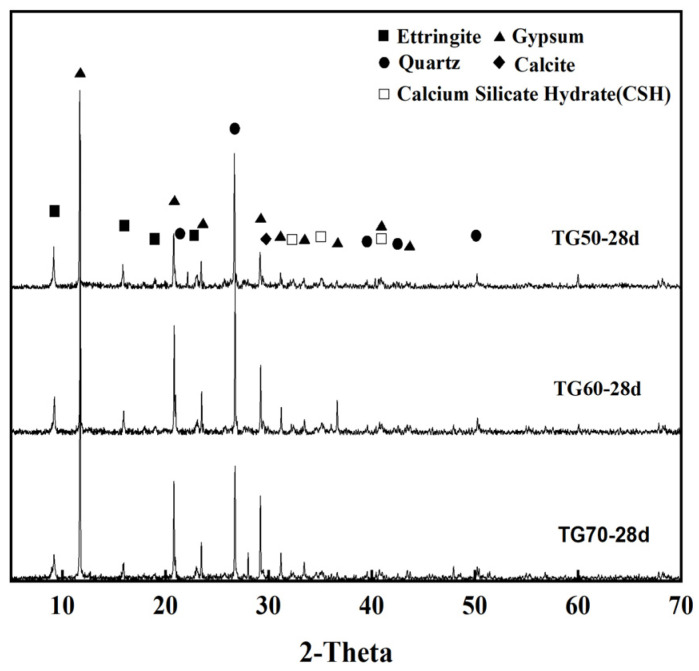
XRD patterns of TG50, TG60, and TG70 at 28 days.

Figure 22 presents the XRD diffraction patterns of samples with TG contents of 50%, 60%, and 70% (i.e., TG50, TG60, and T70) after 28 days of curing. These samples were prepared with a constant water-to-binder ratio of 0.46 and 5% quicklime addition. The XRD analysis reveals that the primary hydration products in the CLSM at 28 days are ettringite, calcium silicate hydrate (C-S-H), and some residual gypsum crystals, which are similar to the results of existing literature [3,38]. It is evident that the gypsum diffraction peaks increase significantly with the rise in TG content, indicating that only a portion of the TG participates in the reaction to form ettringite, while a substantial amount of unreacted TG fills the voids in the system. Concurrently, the diffraction peak of ettringite shows a decreasing trend with the increase in TG content. This trend is attributed to the reduced relative content of GGBS and fly ash, which are the main contributors to the hydration reaction and the formation of ettringite. Consequently, the amount of hydration products formed within the hardened paste decreases, leading to a reduction in the compressive strength of the CLSM samples.

**Figure 23:**
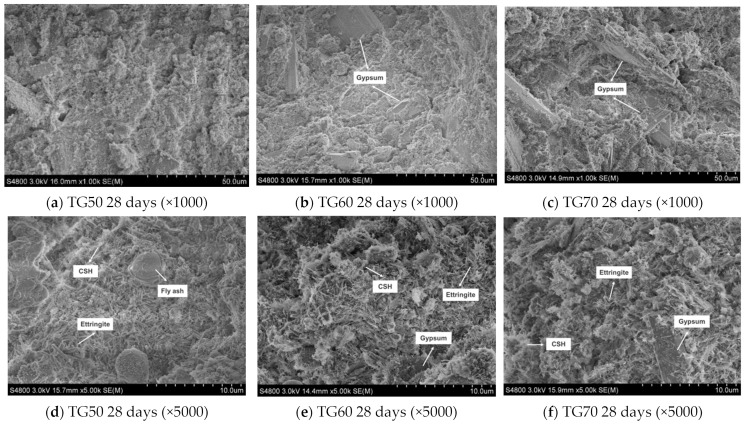
SEM images of TG50, TG60, and TG70 at 28 days.

The SEM images of TG50, TG60, and T70 are shown in Figure 23. It can be observed that the sample with 70% TG content exhibits more porosity and fewer hydration products, such as ettringite, compared to the other two groups. The connections between ettringite, calcium silicate hydrate (C-S-H) gel, and unreacted TG crystals are relatively loose. As the TG content decreases, the relative amounts of GGBS and fly ash increase, leading to a significant rise in the quantity of hydration products in the samples.

#### 3.5.3. The Effect of Quicklime

**Figure 24:**
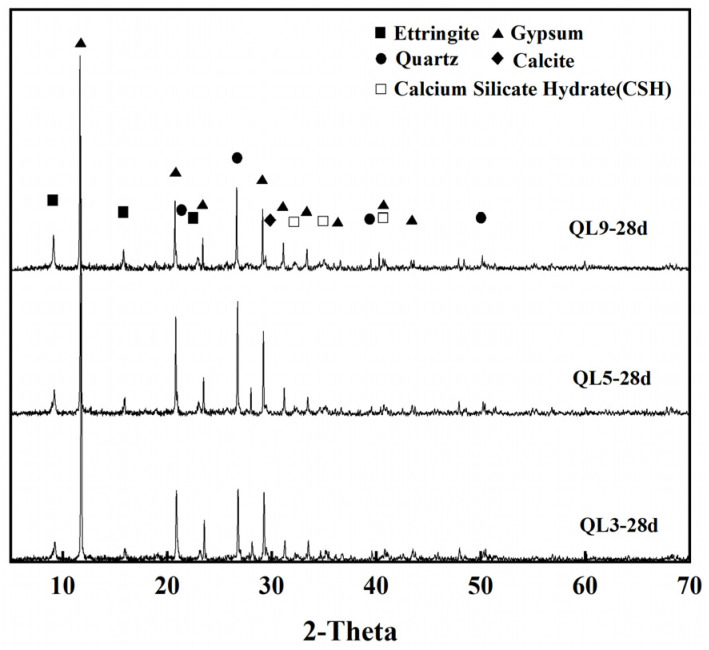
XRD patterns of QL3, QL5, and QL9 at 28 days.

Figure 24 presents the XRD patterns of samples with a fixed content of 70% TG and a water-to-solid ratio of 0.46, incorporating 3%, 5%, and 9% quicklime (i.e., QL3, QL5, and QL9) after a 28-day curing period. It can be observed from the figure that the ettringite diffraction peak intensity at 28 days for the sample group with 3% quicklime is lower than that of the other two groups. This indicates that with a lower quicklime content, the amount of ettringite formed at 28 days is minimal, while the diffraction peak intensity of ettringite in the other two groups increases with the quicklime content. Hence, it is evident that the quantity of the hydration product ettringite is closely related to the quicklime content; the higher the quicklime content, the more ettringite is formed. In the quicklime-activated TG-based CLSM system, due to the continuous excess of SO_4_^2−^ during the hydration process, the formation of ettringite is controlled by the alkalinity, and the concentrations of Ca^2+^ and Al^3+^, with the solubility of ettringite significantly decreasing as pH increases. Therefore, the increase in quicklime content facilitates the formation of ettringite crystals, and the quantity of ettringite formed in the system also reflects the rate of raw material hydration. Additionally, the figure reveals that the diffraction peak intensity of calcite in the sample with 9% quicklime is slightly higher than that of the other two groups, indicating that the increase in quicklime content deepens the carbonation of the CLSM samples. The calcite crystals formed through carbonation fill the intergranular voids, thereby enhancing the density of the pores and, to some extent, increasing the strength.

**Figure 25:**
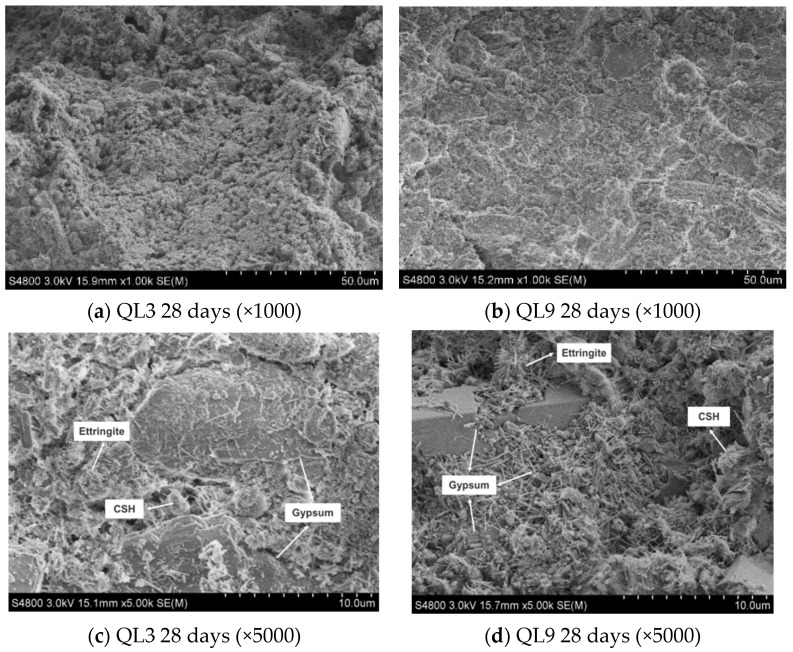
SEM images of QL3 and QL9 at 28 days.

The SEM images of QL3 and QL9 at 28 days are shown in Figure 25, respectively. At 3% quicklime (QL3), the hydration products are mainly ettringite and calcium silicate hydrate gel, but in limited quantities with very fine needle-like ettringite crystals. With 9% quicklime (QL9), the SEM images reveal significant reaction traces on thick gypsum crystals and an increased amount of hydration products. The needle-like ettringite crystals are more abundant and interwoven within the structure, enhancing the paste’s density by connecting agglomerated construction debris particles. This demonstrates that quicklime content is crucial for CLSM strength, as higher alkalinity from quicklime promotes the rapid depolymerization of GGBS and fly ash, releasing [AlO_2_]^−^ ions that form ettringite with Ca^2+^, SO_4_^2−^, and OH^−^. Lower alkalinity slows down the dissolution of these materials, resulting in fewer hydration products and lower strength.

## 4. Conclusions

This study primarily investigates the working and hardening properties of CLSM based on titanium gypsum and construction waste soil and analyzes the effects of various factors on the aforementioned performance characteristics. Additionally, the strength formation mechanism of CLSM was analyzed using XRD and SEM. The conclusions obtained are as follows:

(1) The increase of the replacement rate of CWS as aggregate significantly decreases the flowability of CLSM. Meanwhile, the bleeding rate decreases as well. When the replacement rate is no less than 40%, the bleeding rate can be controlled within 5%. CLSM prepared with a water-to-solid ratio from 0.40 to 0.46 can meet the fluidity requirements. Additionally, the increase in TG content reduces the flowability of CLSM, which is attributed to the relatively low density, large specific surface area, and strong water-absorption properties of TG.
(2) The relationship between bleeding rate and flowability was investigated. The research reveals an approximately positive correlation between them. To ensure that CLSM meets the requirement of a bleeding rate below 5% in practical engineering applications, the upper limit of the flowability should be controlled under 300 mm.
(3) The addition of CWS promotes the early stage strength of CLSM to some extent, yet it restricts the strength development in the later stage. Both an increase in the water-to-solid ratio and the amount of TG reduce the compressive strength of CLSM, while an increase in the amount of quicklime enhances its strength. Notably, when the quicklime content is between 3% and 5%, the 56 day compressive strength is below 2.1 MPa, which is highly favorable for secondary excavation in the later stage.
(4) The volume expansion rate of CLSM continuously decreases with the increase in the replacement rate of CWS. An increase in the water-to-solid ratio raises the expansion rate, while an increase in the TG content significantly reduces it. Specifically, when the TG content is 70%, the CLSM volume shows a shrinkage tendency, with the maximum shrinkage occurring at 14 days and a shrinkage rate of 0.27%. To prevent cracks caused by a significant volume expansion or shrinkage of CLSM, the TG content should be controlled within 60% to 70%. Besides, increasing the amount of quicklime can improve the shrinkage of CLSM and play a certain compensatory role in shrinkage.
(5) The characteristics of the stress-strain curves of CLSM with different TG content were explored. The elastic modulus of CLSM was obtained from the stress-strain curves, ranging from 60.6 MPa to 106.5 MPa. The elastic modulus decreases as the amount of titanium gypsum increases, yet it is higher than that of general subgrade soil fillers.
(6) The XRD and SEM analyses revealed the strength formation mechanism of CLSM made from titanium gypsum (TG) and construction waste soil (CWS). As curing progresses, ettringite and calcium silicate hydrate gel form and grow, enhancing the material’s density and strength. Higher TG content reduces hydration products but fills system voids, decreasing overall strength. Conversely, increased quicklime content raises alkalinity, promoting ettringite formation and calcite carbonation, which fill voids and improve strength. Thus, controlling TG and quicklime content is key to optimizing CLSM’s mechanical properties.

## Figures and Tables

**Table 1:** Chemical composition of main raw materials wt.%.

Raw Material	CaO	SiO_2_	Al_2_O_3_	MgO	SO_3_	Fe_2_O_3_	TiO_2_
Titanium gypsum	27.43	4.34	1.37	0.79	34.42	9.28	5.49
GGBS	36.09	33.06	17.03	7.12	0.56	1.24	0.93
Fly ash	2.92	47.73	27.22	0.39	1.83	1.92	1.73
Construction waste soil	8.42	57.79	17.23	1.73	0.17	5.48	0.94

**Table 2:** Physical properties of CWS.

Proportion	Liquid Limit/%	Plastic Limit/%	Plasticity Index/%	Fine Content/200 #, %
2.7	26.5	15.3	11.2	37.74

**Table 3:** CLSM mix proportion.

Samples	Construction Waste Soil Replacement (S/A)/%	Water-to-Solid Ratio	Titanium Gypsum Content/%	Quicklime Content/%	Binder g/100 g				Fine Aggregate g/100 g		Water/g
					Titanium Gypsum	GGBS	Fly Ash	Quicklime	Nature Sand	Construction Waste Soil	
CS	0	0.40	50	5	50	11.25	33.75	5	100	0	80
CWS20	20	0.40	50	5	50	11.25	33.75	5	80	20	80
CWS40	40	0.40	50	5	50	11.25	33.75	5	60	40	80
CWS60	60	0.40	50	5	50	11.25	33.75	5	40	60	80
W0.40	60	0.40	50	5	50	11.25	33.75	5	40	60	80
W0.42	60	0.42	50	5	50	11.25	33.75	5	40	60	84
W0.44	60	0.44	50	5	50	11.25	33.75	5	40	60	88
W0.46	60	0.46	50	5	50	11.25	33.75	5	40	60	92
TG40	60	0.46	40	5	40	13.75	41.25	5	40	60	92
TG50	60	0.46	50	5	50	11.25	33.75	5	40	60	92
TG60	60	0.46	60	5	60	8.75	26.25	5	40	60	92
TG70	60	0.46	70	5	70	6.25	18.75	5	40	60	92
QL3	60	0.46	70	3	70	6.75	20.5	3	40	60	92
QL5	60	0.46	70	5	70	6.25	18.75	5	40	60	92
QL7	60	0.46	70	7	70	5.75	17.25	7	40	60	92
QL79	60	0.46	70	9	70	5.25	15.75	9	40	60	92

Note: (1) Construction waste soil replacement = *m* (Construction waste soil)/*m* (Fine aggregates), donated as S/A; (2) Water-to-solid ratio = *m* (Water)/*m* (Binder + Fine aggregate), donated as W/(B + F); (3) W0.40 = CWS60, TG50 = W0.46, QL5 = TG70.

**Table 4:** Elastic modulus and unconfined compressive strength test results.

TG Content/%	Elastic Modulus/*E*_50_ (MPa)	Unconfined Compressive Strength (*f*_28d_)/MPa	*E*_50_/*f*_28d_
40 (TG40)	106.5	3.11	34.2
50 (TG50)	81.4	2.55	31.9
60 (TG60)	67.9	2.08	32.6
70 (TG60)	60.6	1.86	32.5

**Table 5:** Peak Strain and Ultimate Strain.

TG Content/%	Peak Strain *ε*_0_/× 10^−3^	Ultimate Strain *ε_u_*/× 10^−3^	$\frac{\varepsilon_{u}}{\varepsilon_{0}}$
40 (TG40)	33.37	40.01	1.20
50 (TG50)	33.12	41.65	1.26
60 (TG60)	31.88	41.13	1.29
70 (TG60)	29.38	40.95	1.39

## Data Availability

The original contributions presented in this study are included in the article; further inquiries can be directed to the corresponding author.
